# Antimicrobial resistance in wildlife: detection of β-lactam/carbapenem resistance genes in European hedgehogs (*Erinaceus europaeus*, Linnaeus 1758) in Italy

**DOI:** 10.1007/s11259-026-11204-5

**Published:** 2026-04-13

**Authors:** A. Di Francesco, D. Salvatore, F. Bertelloni, R. Ceccherelli, C. Lupini, V. V. Ebani

**Affiliations:** 1 Department of Veterinary Medical Sciences (DIMEVET), University of Bologna, 40064 Ozzano dell’ Emilia, BO Italy; 2 Department of Veterinary Sciences, University of Pisa, Pisa, 56124 Italy; 3 Lipu Bird Life Italia, Parma, Italy; 4 Centre for Climate Change Impact, University of Pisa, Pisa, 56124 Italy; 5 Department of Veterinary Medical Sciences (DIMEVET), University of Bologna, Ozzano dell’ Emilia, BO Italy

**Keywords:** Antimicrobial resistance, Antimicrobial resistance genes, β-lactam resistance genes, Carbapenem resistance genes, Hedgehog, *Erinaceus europaeus*, PCR, Italy

## Abstract

In the last decades, increasing interest has turned to wild animals living in close contact with humans, pets or livestock, to evaluate their role as sentinels of environmental antimicrobial resistance pollution or potential reservoirs/vectors of antimicrobial-resistant bacteria/antimicrobial resistance genes. The aim of this study was to investigate the presence of β-lactam (including carbapenem) and colistin resistance genes in intestinal samples from 84 European hedgehogs (*Erinaceus europaeus* L. 1758, Mammalia: Erinaceomorpha) collected in central Italy. Unlike most studies based on bacterial isolates, a culture-independent approach was applied, allowing the detection of antimicrobial resistance genes also in non-culturable bacteria. Total DNA from intestinal fragments and faecal material was investigated by PCRs targeting the *bla*_TEM,_ *bla*_CTX−M_, *bla*_SHV_, *bla*_OXA−48_ and *mcr*-1 genes. PCR positivity for β-lactam/carbapenem resistance genes was observed in 46 (55%) of the 84 tested animals. In 26 of 46 positive samples, two or more resistance genes were detected. No positive detections of the *mcr*-1 gene were obtained. The results confirm the potential role of the hedgehog as an environmental sentinel of antimicrobial-resistant bacteria or antimicrobial resistance genes.

## Introduction

Antimicrobial resistance (AMR) represents one of the major global health threats, by impairing our capacity to treat an increasing number of infections, which leads to economic and social consequences (Vittecoq et al. 2016). Estimates based on European Antimicrobial Resistance Surveillance Network data from 2020 indicate that more than 35,000 people in the European Union/European Economic Area die annually as a direct consequence of antimicrobial-resistant infections (https://www.ecdc.europa.eu/en/publications-data/antimicrobial-resistance-eueea-ears-net-annual-epidemiological-report-2024).

Antimicrobials are produced naturally by strains of bacteria and fungi in all environments, including soil (Allen et al. 2010), thus AMR can be found in many ecosystems, even those that are not anthropised. In this regard, a recent study showed that some specific lineages of methicillin-resistant *Staphylococcus aureus* appeared in European hedgehogs in the pre-antibiotic era (Larsen et al. 2022).

In the last decades, the overuse and misuse of antimicrobials in human and veterinary medicine to improve health and productivity, as well as in the agricultural and industrial fields, increased dramatically the selection pressure for and the prevalence of AMR (Vezeau and Kahn 2024). In the veterinary field, most investigation has focused on intensively reared animals, as they are more exposed to pharmacological pressure and more recently on companion animals. However, in accordance with the One Health concept — recognising the interconnectedness of humans, domestic and wild animals, plants and the wider environment — a growing number of studies considered the potential role of wildlife in the complex phenomenon of AMR (Plaza-Rodríguez et al. 2021). Although wild animals are unlikely to be treated with antibiotics, except occasionally in rehabilitation facilities (Plaza-Rodríguez et al. 2021), antimicrobial-resistant bacteria and/or antimicrobial resistance genes (ARGs) have been detected in numerous wildlife species (Radhouani et al. 2014; Ramey 2021). Multi-drug-resistant strains were more frequently found in wild animals that used anthropised sites due to anthropic AMR pollution of the ecosystems they inhabited (Laborda et al. 2022). Therefore, increasing interest has turned to wild animals living in close contact with humans, pets or livestock to evaluate their role as sentinels of environmental AMR pollution or potential reservoirs/vectors of antimicrobial-resistant bacteria/ARGs (Darwich and Molina-López 2023).

Hedgehogs are extremely adaptable and frequent a wide range of open and vegetated habitats. They are abundant in private gardens and public parks, living in close contact with humans and pets, but also with livestock in the countryside. Hedgehogs are omnivores; their diet includes insects, spiders, snails, earthworms, small amphibians, reptiles, ground-nesting bird eggs, fruits, vegetables, and waste and food scraps (Garcês 2022). Habitat, eating habits, and close contact with humans, pets or livestock can expose hedgehogs to a wide range of sources of AMR bacteria and/or resistance genes, e.g. through contaminated water, soil and food (Garcês 2022). Recent studies highlighted AMR in hedgehogs, mainly in enteric Gram-negative bacteria (Krawczyk et al. 2015; Darwich et al. 2019; Garcias et al. 2021; Dierikx et al. 2023; Jajarmi et al. 2025).

The aim of this study was to investigate the occurrence of antimicrobial resistance genes associated with β-lactam (including carbapenem) and colistin resistance in intestinal samples from European hedgehogs (*Erinaceus europaeus* L. 1758, Mammalia: Erinaceomorpha) from central Italy using a culture-independent PCR-based approach. Although targeted PCR detection of resistance genes in total DNA does not permit attribution to a specific bacterial species and therefore has epidemiological but not diagnostic significance, it avoids potential underestimation of AMR due to non-culturable or slow-growing bacteria (Galhano et al. 2021) and can be useful in environmental AMR surveillance and/or in AMR wildlife investigations, where the advanced decomposition of carcasses could reduce the reliability of culture-dependent methods (Guardabassi and Agerso 2006, Seyfried et al. 2010; Blanco-Peña et al. 2017; Di Francesco et al. 2020; Smoglica et al. 2020; Luo et al. 2022; Di Francesco et al. 2023a, 2023b, 2024).

## Materials and methods

From June 2024 to June 2025, 84 hedgehog carcasses from road accidents in urban and peri-urban areas of the cities of Livorno and Pisa (Tuscany region, central Italy) and collected at CRUMA-LIPU wildlife recovery centre (Livorno, Italy) were submitted to the Department of Veterinary Sciences of the University of Pisa for teaching purposes. During necropsy, fragments of lower gastrointestinal tract and faecal material were collected and stored at − 20 °C until DNA extraction was performed using the QIAamp DNA Mini Kit (Qiagen, Hilden, Germany), following the manufacturer’s instructions. A negative control (kit reagents only) was included in each extraction set. The DNA extracts were stored at − 20 °C and subsequently sent to the Department of Veterinary Medical Sciences at the University of Bologna (Italy) for molecular assays.

The DNA samples were investigated for the presence of the ARGs *bla*_TEM_, *bla*_CTX−M_, *bla*_SHV_ (β-lactam resistance genes), *bla*_OXA−48_ (carbapenem resistance gene), and *mcr-*1 (colistin resistance gene). Each gene was amplified by an individual PCR, according to Jouini et al. (2007; *bla*_TEM_ and *bla*_SHV_), Batchelor et al. (2005; *bla*_CTX−M_), Loucif et al. (2022; *bla*_OXA−48_) and Liu et al. (2016; *mcr*-1). A specific positive control was used for each ARG tested, using DNAs extracted from *Escherichia coli* field strains, containing antimicrobial resistance plasmids. The extraction control and a distilled water negative control were also included. The PCR products were analysed by 2% agarose gel electrophoresis; the DNA bands were stained with Midori Green Advance (Nippon Genetics Europe GmbH, Düren, Germany) and then visualised using ultraviolet trans-illumination. All amplicons were purified using NucleoSpin Gel and PCR Clean-up Mini kit (Macherey-Nagel, Düren, Germany), and both DNA strands were sequenced by the Sanger method (Bio-Fab Research, Rome, Italy). The sequences obtained were compared with publicly available sequences by using the BLAST server in the GenBank database (National Center for Biotechnology Information 2025).

## Results

A PCR positivity was highlighted in 46 (55%; 95% CI: 44–66%) out of the 84 tested animals. In particular, 40 (48%; 95% CI: 37–59%) out of the 84 intestinal samples were positive for *bla*_TEM−1_, 25 (30%; 95% CI: 20–40%) for *bla*_CTX−M_, 7 (8%; 95% CI: 2–14%) for *bla*_SHV_ and 6 (7%; 95% CI: 2–12%) for *bla*_OXA−48_. In contrast, *mcr*-1 gene was not detected in the tested animals (Table 1).

**Table 1:** Prevalence of antimicrobial resistance genes detected in the intestinal samples (*n* = 84) from hedgehogs

ARGs	Positive samples		
	Number	%	95% CI
*bla*_TEM−1_	40	48%	37–59%
*bla*_CTX−M_	25	30%	20–40%
*bla*_SHV_	7	8%	2–14%
*bla*_OXA−48_	6	7%	2–12%
*mcr*-1	0	0%	-

In 26 out of 46 (56.5%) positive samples, 2 or more β-lactam/carbapenem resistance genes were detected (Table 2). The most frequent gene combination was *bla*_TEM−1_ + *bla*_CTX−M_, detected in 23/84 (27%; 95% CI: 17–36%) of the tested samples. In particular, *bla*_TEM−1_ gene (26 vs. 14) as well as *bla*_CTX−M_ gene (23 vs. 2) were more often found in association with other genes than alone. *bla*_SHV_ gene was found exclusively in association, while *bla*_OXA−48_ gene was found more frequently not associated (4 vs. 2).

**Table 2:** Co-occurrence patterns of antimicrobial resistance genes detected in the intestinal samples (*n* = 84) from hedgehogs

Genotypic pattern	Positive samples		
	Number	(%)	95% CI
*bla*_TEM−1_	14	17%	9–25%
*bla*_CTX−M_	2	2%	0–5%
*bla*_SHV_	0	0	0
*bla*_OXA−48_	4	5%	0–10%
*bla*_TEM−1_+*bla*_CTX−M_	18	22%	12–30%
*bla*_TEM−1_+*bla*_CTX−M_+*bla*_SHV_	5	6%	1–11%
*bla*_TEM−1_+*bla*_OXA−48_	1	1%	0–3%
*bla*_TEM−1_+*bla*_SHV_+*bla*_OXA−48_	1	1%	0–3%
*bla*_TEM−1_+*bla*_SHV_	1	1%	0–3%
Without resistance genes	38	45%	34–56%
**Total**	84	100%	-

Amplicon identity was confirmed by comparison between the sequences obtained and the corresponding sequences available in the GenBank database, showing 99%–100% nucleotide similarity. Since the sequences obtained were identical to each other for each of the four amplified ARGs, a representative sequence for each gene was deposited in the GenBank database under accession numbers PX768970-PX768973.

## Discussion

The European hedgehog, also known as the West European hedgehog or common hedgehog, is a synanthropic mammal widely distributed in Europe. In Italy it is present throughout the peninsula, Sicily, Sardinia and some smaller islands (Battisti 2022). The species is protected by the Bern Convention (L. 503/1981) and in Italy by national law 157/1992, which prohibits its hunting, capture and possession.

To our knowledge, few investigations have been performed in Italy regarding the presence of AMR bacteria/ARGs in European hedgehogs. A study on the presence of 14 tetracycline (*tet*) resistance genes in different tissues of European hedgehogs reported that all hedgehogs tested from Italy had at least one resistance gene (Di Francesco et al. 2020). Recently, 39/54 (72.20%) staphylococci isolated from hedgehogs from central Italy showed phenotypic resistance to at least one antimicrobial tested and 21/54 (38.80%) showed more than one resistance type (Bertelloni et al. 2025). In addition, a study of antimicrobial resistance in *E. coli* from European hedgehogs admitted to a wildlife rescue centre in Italy, highlighted that over half of hospitalised animals not previously treated with antibiotics harboured *E. coli* that was resistant to at least one antimicrobial, including extended-spectrum β-lactamases (ESBLs)-producing *E. coli* in three (5.7%) of 53 animals (Prandi et al. 2025).

The aim of this study was to investigate the occurrence of antimicrobial resistance genes associated with β-lactam and colistin resistance in intestinal samples from hedgehogs using a culture-independent molecular approach.

β-lactams are among the most commonly used antibiotics because of their minimal side effects and broad antibacterial spectrum. Resistance to β-lactams continues to increase, posing a public health concern worsened by the rapid evolution of ESBLs, a group of enzymes that confer resistance to most β-lactam antibiotics, including expanded-spectrum cephalosporins and monobactams (Castanheira et al. 2021). The most common types of ESBLs are variants of the TEM, SHV and CTX-M enzymes, resulting from amino acid substitutions that alter their substrate profile (Castanheira et al. 2021).

Carbapenems are broad-spectrum, β-lactam antimicrobials used to treat infections caused by multidrug-resistant bacteria, including β-lactam resistant enterobacteria, and are therefore considered last-resort drugs. In the light of the continuing lack of new antibiotics with anti-Gram-negative activity, carbapenem resistance is a cause of much clinical concern. A recent scientific opinion from European Food Safety Authority (EFSA BIOHAZ Panel 2025) reported that carbapenemase-producing bacteria, once restricted to only hospitals, are now found in food-producing animals, predominantly pigs, their environment and the food chain in the European Union and European Food Trade Association (EU/EFTA). Taking into account that carbapenems have never been authorised for use in food-producing animals in the EU/EFTA, to reserve them for human use only, the presence of carbapenem resistance genes in livestock, as well as in companion and wildlife animals, is suggestive of anthropogenic environmental contamination (Garcias et al. 2021).

Colistin is a polymyxin antibiotic that has been used in veterinary medicine for decades, as a treatment for enterobacterial digestive infections, as well as a prophylactic treatment and growth promoter in livestock, leading to the emergence and spread of colistin-resistant Gram-negative bacteria. To date, several *mcr* gene types encoding enzymes that confer resistance to colistin, primarily *mcr*-1, have been described worldwide in animals, food samples and humans (Luo et al. 2020). The colistin resistance represents a great public health concern, considering that colistin is one of the last-resort antibiotics against multidrug-resistant deadly bacteria, in particular strains resistant to carbapenems (Valiakos and Kapna 2021).

The results of our study showed the presence of β-lactam and carbapenem resistance genes in 46 (55%) of 84 tested European hedgehogs. This percentage is higher than that observed in previous studies performed on bacterial isolates from hedgehogs (Darwich et al. 2019, 26% β-lactam and carbapenem resistance genes; Garcias et al. 2021, 36.8% β-lactam and carbapenem resistance genes; Jajarmi et al. 2025, 42% β-lactam resistance genes). In our study, a molecular, culture-independent approach was applied, amplifying total DNA extracted from intestinal samples. Consequently, resistance genes were amplified by the total bacterial load and not limited to isolates, validating the high percentage of positivity found.

According to Jajarmi et al. (2025), *bla*_TEM_ (48%) and *bla*_CTX−M_ (30%) were the most frequently found ARGs, followed by *bla*_SHV_ (8%). These genes encode serine β-lactamases belonging to class A of the Ambler classification (based on amino acid sequence homology). In the past, TEM and SHV types were the predominant β-lactamases, currently replaced by the CTX-M type that was highlighted in nosocomial and community settings as well as in companion animals, the environment, food products and livestock (Castanheira et al. 2021). In this study, the sequence analysis of the *bla*_TEM_ amplicons obtained did not highlight the amino acid substitutions that are most frequently involved in conferring the ESBL phenotype to TEM-type enzymes, such as those involving Glu104, Arg164, Gly238 and Glu240 (Castanheira et al. 2021). The comparison with the corresponding sequences available in the GenBank database allowed the amplicon to be identified as *bla*_TEM−1_. This result is significant since the resistance conferred by the most common TEM variants, such as TEM-1, is not related to Critically Important Antimicrobials (CIA) that are, according to the World Health Organization, antibacterial agents crucial for treating severe human infections, e.g. third-generation cephalosporins, quinolones, polymyxins. Indeed, TEM-1 enzyme primarily confer resistance to penicillins and ampicillin (Bradford 2001), which are not classified as CIA.

With respect to SHV-β-lactamases, the majority of SHV variants possessing an ESBL phenotype are characterized by the substitution of a serine for glycine at position 238 (Bradford 2001). Unfortunately, the SHV sequences obtained in this study were too short to detect the potential amino acid substitution or assign a subtype.

A similar consideration could also be made for the CTX-M sequences, although in this case the length of the sequence is less discriminating, since the extended-spectrum activity of the CTX-M-type appears to be related to the serine residue at position 237, which is present in all of the CTX-M enzymes (Bradford 2001).

Interestingly, six (7%) of the 84 European hedgehogs that we sampled were PCR positive for the bla_OXA−48_ gene, encoding OXA-48 carbapenemase, belonging to class D of the Ambler classification. It is currently the most common global OXA-48-like enzyme. *Enterobacterales* with OXA-48 have been detected from humans, animals (companion, farm, and wildlife) and various types of environmental specimens from different parts of the world (Mairi et al. 2018).

β-lactamase genes frequently co-occur in clinical and environmental bacterial isolates. In this study, 26 out of 46 (56.5%) positive samples showed 2 or more β-lactam/carbapenem resistance genes. According to Jajarmi et al. (2025) the most frequent gene combination was *bla*_TEM−1_ + *bla*_CTX−M_, detected in 23/84 (27%) of the tested samples. Otherwise, Garcia et al. (2021) reported that the combination *bla*_CTX−M−15_ with *bla*_SHV−28_ was the most repeated association found.

All these genes are often found on plasmids, transposons, insertion sequences that could contribute to their dissemination between various bacterial species via horizontal transmission.

In our study, no samples were positive for *mcr*-1 colistin resistance gene. This negative result was not surprising, considering that in Italy the use of colistin is closely monitored in both human and veterinary medicine to limit antimicrobial resistance, with significant restrictions on prophylaxis and use in combination with other drugs. In the veterinary field, colistin is used mainly for the treatment of intestinal infections caused by Gram-negative bacteria in food-producing animals. The lack of evidence of *mcr*-1 gene could be attributed to the urban origin of the hedgehogs tested in our study and to the limited possibility of their contact with livestock. In this regard, Garcias et al. (2021) reported negative *mcr* results in hedgehogs from urbanised areas of Catalonia (Spain).

Our results confirm the potential role of the hedgehog as a sentinel species of environmental AMR contamination, for reasons that also make it a good bioindicator of metal and pesticide pollution (wide distribution, urban or agricultural habitat, feeding habits) (García-Muñoz et al. 2025). A limitation of this study is the poor sample representativeness, since only hedgehogs from two urban areas were examined which restricts the generalisability of the results to the national population and did not allow an urban vs. rural comparison. Added to this is the lack of direct comparisons with environmental samples, domestic animals, or humans, that does not allow us to identify undoubtedly the origin of the AMR contamination. Nonetheless, we believe that our study can provide a useful contribution to the study of AMR in wildlife, taking into account the limited availability of data from Italy on the presence of antibiotic resistance genes of critical importance to human medicine in wild mammals.

Regarding the role of wildlife as a reservoir or vector for the spread of AMR, it should be explored further through active surveillance, comparing bacteria isolated from wildlife with those from humans (Dierikx et al. 2023) or domestic animals, as well as conducting longitudinal sampling over extended periods to assess the persistence and dissemination of AMR by wildlife (Dolejska and Literak 2019; Zeballos-Gross et al. 2021). Given that ARGs are often found on mobile genetic elements able to move within and between bacterial species, thereby contributing to their dissemination and that hedgehogs can harbour many bacteria, including zoonotic pathogens (Ruszkowski et al. 2021; Đuričić and Lukač 2025), the role of hedgehog as an AMR reservoir deserves to be explored further.

## Conclusions

In conclusion, the results of this study agree with previous reports suggesting the role of wildlife as vector or bioindicator of resistant bacteria and genetic determinants of antimicrobial resistance in the environmental compartment, of which wildlife is a part.

Further studies would be desirable in order to investigate in more depth the potential role of wildlife as a reservoir of AMR, in order to better safeguard human, animal and environmental health, in a One Health perspective.

## Data Availability

All data used and/or analysed during this study are included in this article and are available from the corresponding author on reasonable request. The sequences generated in this study are available in GenBank under accession numbers PX768970- PX768973.
